# Hemorrhagic shock and fluid dynamics

**DOI:** 10.14814/phy2.14813

**Published:** 2021-03-26

**Authors:** Maya Cohen, Sean F. Monaghan

**Affiliations:** ^1^ Division of Pulmonary Critical Care, and Sleep Medicine Department of Medicine Alpert Medical School of Brown University /Rhode Island Hospital Providence RI USA; ^2^ Division of Surgical Research Department of Surgery Alpert Medical School of Brown University/Rhode Island Hospital Providence RI USA

Hemorrhagic shock is a type of hypovolemic shock in which acute blood loss results in inadequate delivery of oxygen to the tissues and cells. Hemorrhage is a major cause of morbidity and mortality, contributing to more than 60,000 deaths per year in the United States and approximately 1.9 million deaths per year worldwide (Lozano et al., 2012). Traditional treatment has focused on controlling hemorrhage and restoring macrovascular parameters, such as mean arterial pressure and hemoglobin concentration, via blood and fluid infusions and vasopressor use. However, even with normalization of systemic hemodynamics, a portion of patients still go on to develop multiple organ dysfunction.

The reason for this persistent dysfunction is complex and still being probed. Both animal and human models have shown that the microvasculature can remain impaired for up to 72 hours after the original hemorrhage and resuscitation events (Hutchings et al., 2018; Tachon et al., 2014). Some of the underlying molecular mechanisms have been elucidated. For example, in response to hemorrhagic shock, the endothelial glycocalyx barrier breaks down, contributing to subsequent fluid and solute extravasation. Additionally, a posthemorrhage inflammatory response occurs, with various cytokines and chemokines affecting leukocyte migration, coagulation changes, and vasomotor alterations. Reactive oxygen species also form after immune cell activation. And therapeutic strategies themselves can have deleterious effects, such as when aggressive volume resuscitation causes further microvascular stress and tissue edema. (Torres Filho, 2017).

In this issue of *Physiologic Reports*, Jani et al investigate the mechanics of the microvasculature in response to hemorrhagic shock (Jani et al.,). As explained in their article, the microcirculation is primarily controlled by Starling forces:

$$\text{Jv}=\text{Kf}\left[\left(\text{Pc}‐\text{Pi}\right)‐\left(\pi c‐\pi i\right)\right]$$

Net fluid movement across capillaries (Jv) is determined by the sum of capillary pressures (hydrostatic, Pc; and oncotic, πc) and interstitial pressures (hydrostatic, Pi; and oncotic, πi) (Levick & Michel, 2010). The current treatments for shock—volume infusions, vasopressors—ultimately modulate the capillary hydrostatic and oncotic pressures. Here, Jani et al examine how manipulation of the interstitial hydrostatic pressure might affect microvascular dynamics.

The investigators outfitted hamster models with a dorsal window chamber and a custom‐designed negative pressure application device via intravital microscopy. They then simulated class IV hemorrhagic shock with 40% total blood volume loss via a carotid artery catheter. There were three animal cohorts: those with (a) negative pressure application during normovolemia, (b) negative pressure application during hypovolemia, and (c) no negative pressure application during hypovolemia. The measured endpoints included arteriolar and venular diameter changes, arteriolar and venular blood flow (as calculated using Poiseuille's law), and functional capillary density (FCD). That last endpoint is defined as the number of capillaries that possess transiting RBCs, and this was the only endpoint correlated with survival posthemorrhagic shock (Kerger et al., 1996).

The authors found that application of negative pressure during hypovolemia did initially improve FCD (0.66 ± 0.02) compared to the nonnegative pressure group (0.50 ± 0.04); though it did not normalize it, and statistically significant differences were lost after 90 minutes. Additionally, negative pressure application during both hypovolemia and normovolemia improved mean capillary perfusion pressure as indicated by increased venular outflow. Interestingly, in larger arterioles (>40 μm), negative pressure application during hypovolemia *decreased* flow compared to hypovolemia alone; but this did not seem to impact downstream FCD or intramural capillary pressure. The authors hypothesized that this difference could be due to endogenous mediators of vasoconstriction, which would have greater effect on the arterial resistance vessels than the venous capacitance vessels.

This study has significant clinical implications. The authors elegantly demonstrated that modulation of interstitial hydrostatic pressure can improve perfusion in hemorrhagic shock without fluid resuscitation. While volume resuscitation is an essential part of treatment for the hemorrhagic patient, it does not necessarily account for the physiologic and molecular changes at play in the microcirculation. Additionally, aggressive volume resuscitation has known deleterious side effects including edema formation, acidemia, hypothermia, and hemodilution that reduces the oxygen‐carrying capacity of blood (Leeuwen et al., 2020). The hope is that new treatment modalities might attenuate our reliance on massive volume resuscitation and blood transfusions.

As with all research studies there are methodology limitations and future questions to be answered. Animal models always raise the issue of applicability to human physiology. Moreover, the authors’ negative pressure device only targeted the skeletal muscle and subcutaneous tissue circulations; applying this concept to other organ systems—with heterogeneous physiologies and varying accessibility—is more theoretical than practical at this point (Govender et al., 2021). Lastly, this experiment isolated the effects of one treatment modality. In clinical practice multiple treatments are started at once for the crashing shock patient. Future studies are needed to tease out the effects of simultaneous negative pressure application, volume resuscitation, and vasopressor usage.

In summary, this study by Jani et al. provides physiologic insight into microvascular dysfunction in hemorrhagic shock, as well as a potential new treatment modality. Their novel finding—that applied negative interstitial pressure improves microvascular perfusion in an animal model—raises questions about application to human clinical contexts and lays the groundwork for future investigations.
